# *Drosophila* CP190- and dCTCF-mediated enhancer blocking is augmented by SUMOylation

**DOI:** 10.1186/s13072-017-0140-6

**Published:** 2017-07-04

**Authors:** Theresa Jox, Melanie K. Buxa, Dorte Bohla, Ikram Ullah, Igor Mačinković, Alexander Brehm, Marek Bartkuhn, Rainer Renkawitz

**Affiliations:** 1 Institute for Genetics, Justus-Liebig-University, 35392 Giessen, Germany; 2 Institute of Molecular Biology and Tumour Research, Philipps University Marburg, 35037 Marburg, Germany; 3 Institute for Molecular Pathology, UKGM, 35392 Giessen, Germany; 4 Flohr Consult, Adenauerallee 136, 53113 Bonn, Germany

**Keywords:** Chromatin insulator, CP190, dCTCF, SUMOylation, Fab8, Insulator speckle

## Abstract

**Background:**

Chromatin insulators shield promoters and chromatin domains from neighboring enhancers or chromatin regions with opposing activities. Insulator-binding proteins and their cofactors mediate the boundary function. In general, covalent modification of proteins by the small ubiquitin-like modifier (SUMO) is an important mechanism to control the interaction of proteins within complexes.

**Results:**

Here we addressed the impact of dSUMO in respect of insulator function, chromatin binding of insulator factors and formation of insulator speckles in *Drosophila*. SUMOylation augments the enhancer blocking function of four different insulator sequences and increases the genome-wide binding of the insulator cofactor CP190.

**Conclusions:**

These results indicate that enhanced chromatin binding of SUMOylated CP190 causes fusion of insulator speckles, which may allow for more efficient insulation.

**Electronic supplementary material:**

The online version of this article (doi:10.1186/s13072-017-0140-6) contains supplementary material, which is available to authorized users.

## Background

Insulators are DNA regulatory sequences that prevent functionally distinct chromatin domains from improper interference. They have basically two functions: the enhancer blocking and the barrier function [1, 2]. The insulator operates as a barrier between chromatin domains. Consequently, spreading of heterochromatin into euchromatin and vice versa is blocked [3, 4]. On the other hand, insulators mediate enhancer blocking by impeding enhancers and silencers from activating or silencing a given promoter. This seems to be mediated by preventing the interaction of both sequence elements [5]. Insulators mediate their function by insulator-binding proteins (IBPs), which bind directly to the insulator sequence and are able to recruit cofactors, like cohesin and remodeling complexes [2]. The highly conserved IBP factor CTCF has been shown to mediate insulation in vertebrates as well as in *Drosophila* [6–11]. A total of nine IBPs have been described in *Drosophila*, such as Su(Hw) [12], BEAF-32 [13–15], Zw5 [16], GAF [17] and the newly identified factors Pita and ZIPIC [18], as well as insulator-binding factors Ibf1 and Ibf2 [19].

In *Drosophila,* dCTCF binds to six out of eight boundary elements of the bithorax complex (BX-C) and thereby contributes to the correct expression pattern of the homeotic genes in this gene cluster [10, 20]. One well-studied, dCTCF-bound insulator in this region is the Fab8 insulator [10, 21]. dCTCF, together with the cofactor CP190, mediates enhancer blocking at this site. CP190 has been found to bind to all nine IBPs of *Drosophila* and to mediate insulator function [2]. This finding by itself does not explain the molecular mechanism of insulation or enhancer blocking. Previously, we therefore performed an RNAi screen to identify additional cofactors required for insulation [22]. We used the Fab8 sequence to insulate a luciferase reporter gene in *Drosophila* S2-cells. Genome-wide RNAi depletion identified many factors required for Fab8-mediated insulation. Among these were the remodeling complexes NURF and dREAM, but also the histone variant H3.3, which have been functionally tested to contribute to insulation [22, 23]. One additional group of proteins identified consisted of factors, which are involved in the SUMOylation cascade. SUMOylation is a modification by small proteins of <20 kDa, comparable to ubiquitination. There are different variants of SUMO in mammals (SUMO-1, 2, 3 and 4), but only one in *Drosophila,* Smt3 [24, 25]. SUMO modification is covalently attached to a specific SUMO motive within the sequence of the target protein [26–28]. Many proteins in different cellular processes are SUMOylated. In *Drosophila*, SUMOylation is known to be required for the regulation of transcription by modifying transcriptional regulators, such as Mi-2, Groucho, Vestigal, CP190 and Mod(mdg4) [29–33]. The role of SUMOylation in insulation and chromatin conformation in *Drosophila* has been controversially discussed. On the one hand, the IBP cofactors CP190 and Mod(mdg4) were found to be SUMOylated [29] and the SUMO modification pathway was shown to antagonize the activity of the *gypsy* insulator [29]. On the other hand, SUMOylation was published to stimulate *gypsy*-mediated insulation by reducing the amount of Mod(mdg4) required for the activity of Su(Hw)-dependent insulation [34].

To get more insight into the role of SUMOylation in the context of insulation and the function of IBPs in *Drosophila*, we expressed FLAG-Smt3 (FLAG-dSUMO) in *Drosophila* S2-cells. We find a striking co-localization of CP190 sites with SUMO and an increase in CP190 chromatin binding upon FLAG-dSUMO expression. SUMO depletion results in a loss of enhancer blocking activity and an increase in insulator speckle formation. Therefore, we can conclude that in the context of an enhancer blocking activity SUMOylation is required.

## Results

### SUMOylation increases enhancer blocking in *Drosophila* S2 cells

**Fig. 1:**
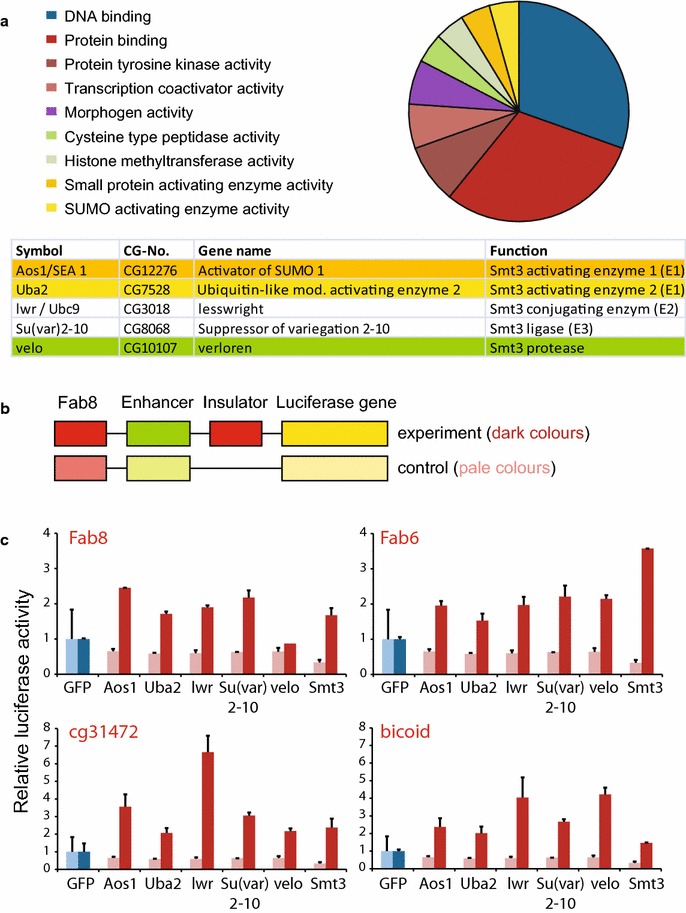
SUMOylation increases enhancer blocking. **a** A functional siRNA screen identified many factors involved in chromatin function [22]. The diagram was generated by the online go term tool GeneCoDis3 listing all factors changing enhancer blocking with a significant *p* value (<0.05). A sub-group of factors (table, *color code* as in **a**) has a function in SUMOylation. **b** Schematic illustration of constructs used to validate SUMO-dependent enhancer blocking. Constructs with insulator (*dark colors*) and control constructs without insulator (*pale colors*) contain the Fab8 insulator for blocking *cis*-regulatory sequences at the integration site (*red*), an enhancer (*green*), the respective insulator (*red*) at the enhancer blocking position (Fab8, cg31472, bicoid, Fab6) and the luciferase gene (*yellow*). **c** Relative luciferase activity after knockdown of SUMOylation factors (*red*) in S2-cells with stably integrated reporter (*dark colors* as in **b**) or control constructs (*pale colors* as in **b**). GFP knockdown (*blue*) was used for normalization

As indicated from a Fab8-mediated enhancer blocking assay carried out previously [22], components involved in the SUMOylation cascade might be involved in the CP190- and dCTCF-mediated Fab8 insulation. In addition to the known chromatin components, factors of the SUMOylation pathway were identified (Fig. 1a). The proteins known to contribute to the SUMOylation activity in *Drosophila* are the dSUMO peptide Smt3, Activator of SUMO-1 (Aos1) and Ubiquitin activating enzyme 2 (Uba2), which form the hetero-dimer of the SUMO-activating enzyme [35, 36], lwr (lesswright or Ubc9), which is the SUMO conjugating enzyme [37] and Su(var)2–10 (suppressor of variegation 2–10), which is related to the conserved PIAS (protein inhibitor of activated STAT) proteins in other organisms and which function as SUMO ligases [38]. Furthermore, the SUMO protease velo (verloren) is required for SUMO function as well [39]. In order to test the functional impact of each of these factors on enhancer blocking, we used a set of reporter constructs (Fig. 1b). These constructs consist of the Fab8 insulator flanking the OpIE2 enhancer, which activates the luciferase reporter gene [22, 40]. The insulator at this position helps to block interference from flanking *cis*-regulatory elements in the genome. A second Fab8 or other insulator sequences were inserted in-between the enhancer and the promoter to block the enhancer activity. A corresponding control construct was lacking a functional insulator sequence at this position. Constructs were transfected into S2 cells, and stable clone pools were generated. These were challenged with double-stranded RNA directed against the known components of the SUMOylation pathway or, as a negative control, against GFP. Depletion of each of the six SUMO controlling factors resulted in an increase in luciferase activity of the Fab8 insulator construct (Fig. 1c). The control reporter construct lacking an insulator sequence at the enhancer blocking position (pale colors in Fig. 1b, c) was not affected, nor did the siRNA against GFP cause unspecific effects. This suggests that optimal enhancer blocking activity mediated by the Fab8 insulator requires the SUMOylation machinery.

To further analyze the functional effect of SUMOylation on enhancer blocking in general, different insulators in addition to Fab8 were tested: cg31472, bicoid and Fab6. These insulators show all dCTCF- and CP190-mediated insulation [10, 22]. Again, stable clone pools were generated with each of these constructs and tested for sensitivity against the depletion of the SUMO factors. Similar to the Fab8 construct, depletion of each of the SUMO components resulted in an increase in gene activity of the constructs with the insulators Fab6, cg31472 and bicoid (Fig. 1c).

In conclusion, enhancer blocking of all four tested insulators is impaired after knockdown of the factors of the SUMOylation cascade. This means that SUMOylation is required for efficient enhancer blocking activity of insulators in *Drosophila* S2 cells.

### FLAG-dSUMO expression SUMOylates *Drosophila* S2 proteins including CP190

**Fig. 2:**
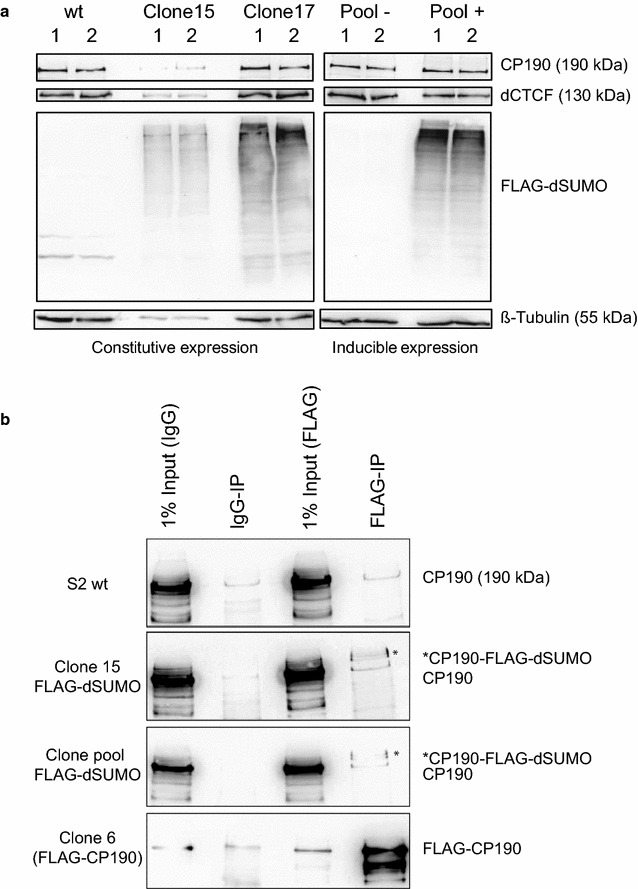
CP190 is SUMOylated. **a** Western blot of extracts from FLAG-dSUMO expressing cells [constitutive clones 15 and 17 and the induced clone pool (+)] detects many SUMOylated proteins with an antibody against FLAG. Control antibodies against CP190, dCTCF and beta-tubulin detect similar ratios between these factors in all cases. S2 wt cells and the non-induced clone pool (−) were used as negative controls. **b** Immune precipitation with the FLAG antibody (FLAG-IP) tested by Western blot with the CP190 antibody identifies SUMOylated CP190 (*) in FLAG-dSUMO expressing cells (CuSO_4_-induced clone pool, clone 15). S2 wt cells and IgG-IP were used as negative controls. Extract from a FLAG-HA-CP190 expressing S2-cell clone (clone 6) served as positive control for the FLAG-IP

In order to study the effect of SUMOylation on insulation mechanistically, we used the FLAG-dSUMO expression system. This system allows improving the efficiency to detect SUMOylated proteins [41]. FLAG-tagged dSUMO was stably expressed in S2 cells, both by constitutive and by inducible expression vectors. With the constitutive expression vector, we generated two cell clones (clones 15 and 17; Fig. 2a). This system was used for knockdown experiments. The inducible expression is driven by a metallothionein promoter and can be used to compare effects in the absence or presence of FLAG-dSUMO. The clone pool generated was devoid of FLAG-dSUMO expression in the absence of the inducing CuSO_4_ and strongly inducible after CuSO_4_ addition (Fig. 2a). A large range of proteins was bound by FLAG-dSUMO as detected by a FLAG antibody. None of the expression conditions did change the amount/ratio of the insulator proteins dCTCF and CP190 and of the control protein beta-tubulin.

To test whether CP190 is SUMOylated, FLAG-immunoprecipitation (FLAG-IP) was performed. As a positive control for FLAG-IP, we used a S2 cell clone expressing a FLAG-HA-CP190 fusion. The Western blot showed that CP190 is SUMOylated (Fig. 2b). A signal for SUMOylated CP190 was detectable with protein extract from the constitutive clone 15 and the induced clone pool. We tested the FLAG-IP material for the presence of dCTCF, but could not detect SUMOylated dCTCF (data not shown). These findings are in line with previous results [29, 30].

### Genome-wide binding strength of CP190 is correlated with and dependent on dSUMO

**Fig. 3:**
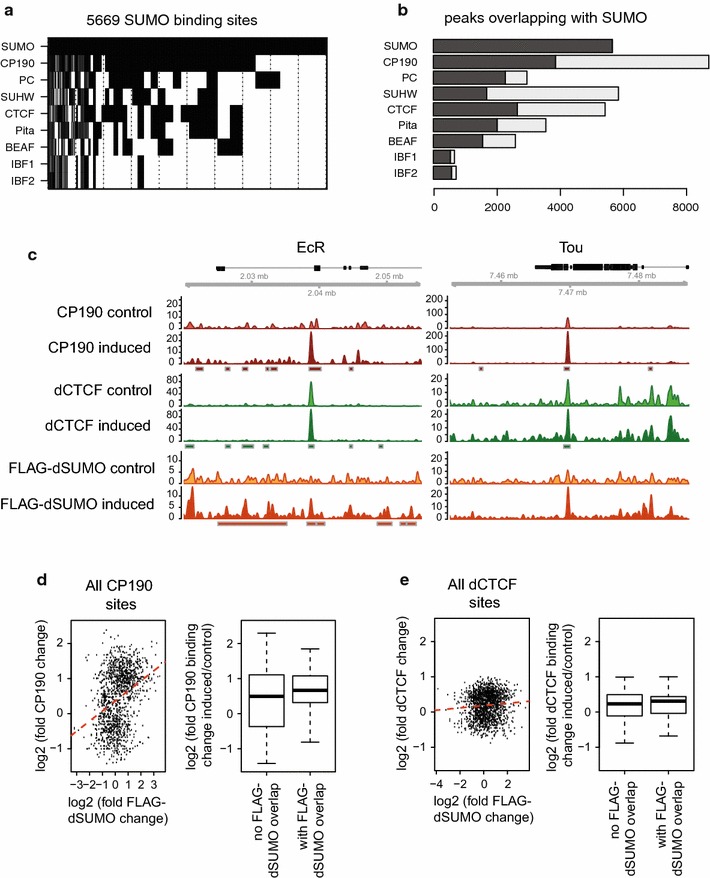
dSUMO co-localizes with insulator-binding proteins and increases CP190 binding in S2 cells. **a** Binary heat map of dSUMO-bound sites (5671 dSUMO peaks), classified for their overlap with CP190, PC, Su(Hw), dCTCF, Pita, BEAF-32 and IfB1 binding [18, 41, 42]. Binding is indicated by *black bars*. The binding sites were sorted for frequency of the binding patterns. **b** The *bar plot* shows the fraction of all binding sites of the indicated factor shared with dSUMO (*black*). The fraction of binding sites not overlapping with dSUMO is shown in *light gray*. **c** Genome browser snap shots of example sites (EcR and Tou) for increased CP190 binding after FLAG-dSUMO expression. CP190 binding (*brown*); dCTCF binding (*green*); FLAG-dSUMO binding (*yellow*); CP190 referent peaks (*red squares*, [42]); dCTCF referent peaks (*green squares*, [42]); dSUMO referent peaks (*yellow squares*, [41]); genes (*black*); before (control) and after induction (induced) of FLAG-dSUMO expression (*pale* and *dark colors*). **d** Genome-wide correlation of log2 fold change in CP190 binding with FLAG-dSUMO binding after induction (*left*); Genome-wide significant (*p* = 8 × 10^−4^) log2 fold binding changes of CP190 with and without FLAG-dSUMO binding (*right*, box blot). **e** Genome-wide analyses of dCTCF binding before and after FLAG-dSUMO expression, analyzed as in (**d**) with a nonsignificant difference (*p* = 0.64)

After the finding that the enhancer blocking function of insulators is dependent on SUMOylation and that CP190 is SUMOylated, we wanted to analyze the genome-wide binding of CP190 in response to changes of the SUMO content within the cells. First we determined the correlation of SUMO-bound chromatin sites with insulator-binding proteins such as dCTCF [11], Pita and ZIPIC [18], BEAF-32 [13–15], Su(Hw) [12] and Ibf1/2 [19]. As a positive control, we also included the binding distribution of Polycomb group proteins (PC), which are known to co-localize with dSUMO [41]. Therefore, we compared ChIP-Seq data of those proteins, which were already available at public data bases [18, 19, 41, 42]. A comparative cluster analysis of binding sites of all factors with dSUMO showed that dSUMO co-localizes at more than half of the binding sites with CP190 (Fig. 3a) . When testing all CP190 sites, again about 50% of the CP190 sites show an overlap with dSUMO (Fig. 3b). In addition, an overlap with dCTCF, PC and Pita is detectable. PC shares nearly all of its binding sites with dSUMO, whereas dCTCF and Pita share about 30–50% of the binding sites with dSUMO. Ibf1 and Ibf2 nearly completely overlap with dSUMO-binding sites (Fig. 3b), whereas BEAF-32 shared half of all binding sites with dSUMO. Su(Hw) showed the lowest co-localization with dSUMO (Fig. 3a, b). Thus, CP190 and its known interaction partners, dCTCF, Pita, BEAF-32, Ibf1 and Ibf2 co-localize strongly with genomic sites bound by dSUMO.

To further analyze the role of SUMOylation for CP190 and dCTCF binding, we used the copper sulfate-induced clone pool expressing FLAG-dSUMO as well as the same clone pool in the absence of copper sulfate. We performed genome-wide ChIP-Seq analyses for dCTCF, CP190 and FLAG-dSUMO. A similar strategy to express FLAG-dSUMO has been used for the analysis of the co-localization of dSUMO with polycomb repressive complexes [41]. The induced situation was compared to the non-induced case to determine any changes in binding upon the expression of FLAG-dSUMO. The ChIP-Seq data showed a comparable binding pattern to published ChIP-Seq data. Both, with or without FLAG-dSUMO expression, dCTCF and CP190 ChIP-Seq signals cluster over published dCTCF or CP190 sites (Fig. 3c; Additional file 1: Figure S1). As expected, FLAG-dSUMO binding was increased after induction (Fig. 3c, purple) when compared to the non-induced control (orange) as exemplified at individual sites in the genome, such as in the EcR and the Tou gene regions (Fig. 3c). CP190 binding was increased after induction of FLAG-dSUMO expression (dark red) in comparison with the control (light red), whereas dCTCF binding remained unchanged upon FLAG-dSUMO expression (dark green) compared to the control (light green: Fig. 3c). The location of these peaks was similar to already published data of dCTCF and CP190 binding (Fig. 3c boxes in green and red; Additional file 1: Figure S1) [42].

Upon FLAG-dSUMO induction, the overall binding of CP190 was strongly changed for most CP190 sites found in the ChIP-Seq experiment. Overall, there was an increase of CP190 binding when comparing sites with and without binding of FLAG-dSUMO (Fig. 3d, box-plot; Additional file 1: Figure S1). Furthermore, there was a positive correlation such that the binding increase was stronger at sites with strong FLAG-dSUMO binding (Fig. 3d, scatter plot). When analyzing the overall changes in dCTCF binding, we found that dCTCF binding strength at its binding sites was nearly unchanged after induction of FLAG-dSUMO expression. This was true at sites with FLAG-dSUMO binding and at sites without FLAG-dSUMO binding (Fig. 3e; Additional file 1: Figure S1).

**Fig. 4:**
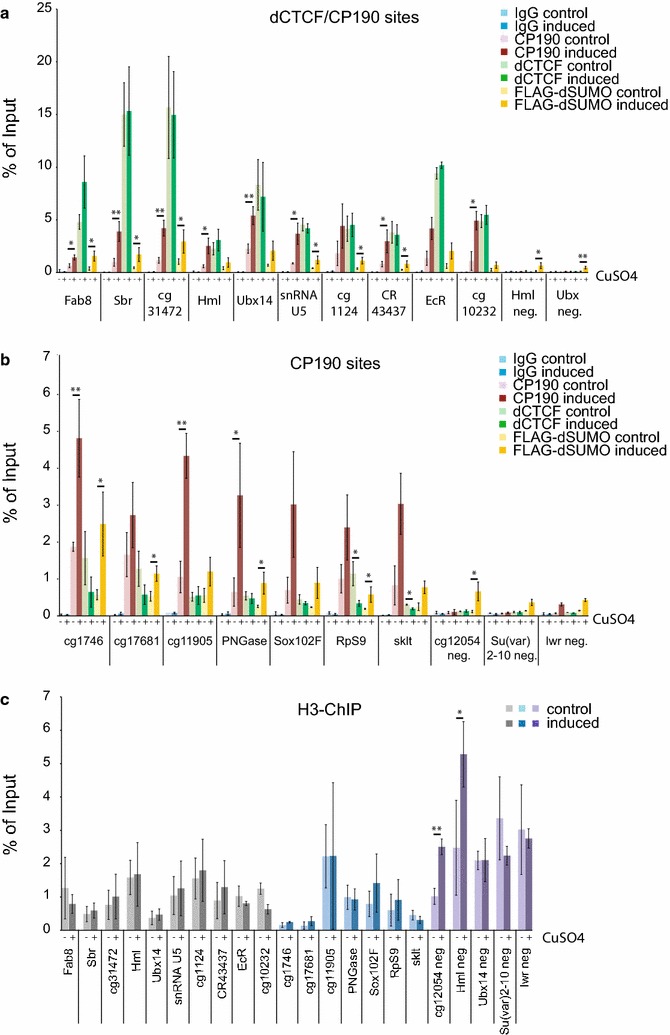
Binding of CP190 is increased after FLAG-dSUMO expression. **a** Binding of CP190 (*red*), dCTCF (*green*) and FLAG-dSUMO (*yellow*) before and after induction of FLAG-dSUMO expression (*pale* and *dark colors*) after ChIP-qPCR at dCTCF/CP190 example sites, in % of input. IgG was used as negative control (*blue*). Sites without any binding of dCTCF and CP190 (Hml neg. and Ubx neg.) were used as control sites. **b** Binding after ChIP-qPCR at CP190 sites devoid of dCTCF binding. Color code as in (**a**), cg12054 neg., Su(var)2–10 neg. and lwr neg. were used as negative controls devoid of dCTCF and CP190. **c** Binding of histone H3 after ChIP-qPCR before and after induction of FLAG-dSUMO expression (*pale* and *dark colors*) at dCTCF/CP190 (*gray*) sites, at CP190 (*blue*) sites and at negative sites (*purple*). Statistical analysis with two-tailed *t* test for significant (*p* value <0.05) changes of binding

For validation, we used the inducible clone pool to analyze CP190, dCTCF and FLAG-dSUMO binding and compared induced with non-induced cells. First, we selected sites, which have been identified as dCTCF/CP190 and CP190 sites, such as Fab8, Sbr, cg31472, Hml, Ubx14, cg1746, cg17681 and cg11905 [10, 18, 21, 22]. Again, a striking co-localization of FLAG-dSUMO and CP190 was detectable (Fig. 4a, b). When CP190 and dCTCF binding was compared between the induced and non-induced cells, CP190 binding was significantly increased, whereas the binding of dCTCF was nearly unaffected (Fig. 4a, b).

As identified in the ChIP-seq results, the top changed sites for CP190 binding after FLAG-dSUMO induction were snRNAU5, cg1124, CR42427, EcR, cg10232 and PNGase, Sox102F, RpS9 and sklt. These showed also in ChIP-qPCR strong changes in CP190 binding after induction of FLAG-dSUMO expression. Again, dCTCF nearly always stayed unchanged after dSUMO-FLAG induction (Fig. 4a, b). Negative sites, with no CP190 or dCTCF binding (cg12054 neg., Hml neg., Ubx neg. and Su(var)2–10 neg.), showed no increase in binding after FLAG-dSUMO induction.

To exclude that FLAG-dSUMO expression has a general effect on the chromatin status, we performed H3-ChIP. No significant changes were detectable in H3 occupancy at nearly all tested CP190 bound sites. Only two of the tested negative sites show a significant change of H3 (Fig. 4c). Similarly, analysis by FAIRE assay could not detect significant changes in chromatin accessibility (data not shown). As an additional verification of SUMO-augmented CP190 chromatin binding, we depleted SUMO by RNAi against Smt3 (dSUMO) or by depletion of both components of the E1 SUMO-activating enzyme, Aos1 and Uba2. In both cases, CP190 chromatin binding was diminished to background levels (Additional file 2: Figure S2).

These findings lead us to conclude that CP190 binding is increased after SUMOylation. This is in agreement with the results of the functional reporter assay above. SUMOylation induces or mediates an increase in binding affinity of CP190 to chromatin. Thereby, enhancer blocking is increased at CP190 bound insulators.

### CP190 speckle number is reduced upon FLAG-dSUMO expression

**Fig. 5:**
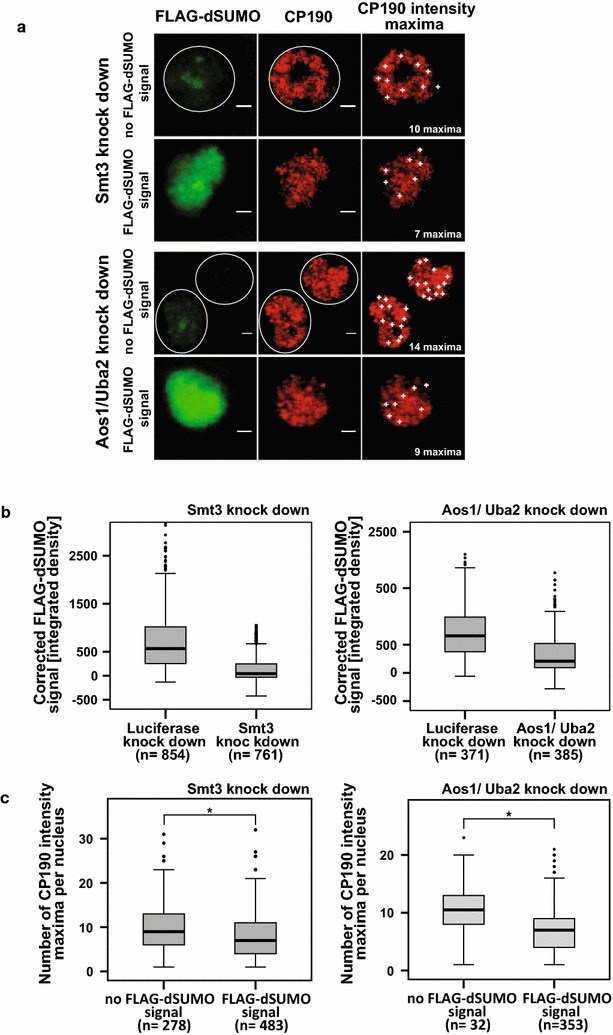
CP190 speckle number is reduced upon FLAG-dSUMO expression. Constitutively FLAG-dSUMO expressing cells were treated with dsRNA against Smt3 or against Aos plus Uba2 (Aos/Uba2). In both cases, cells were stained with antibodies against FLAG (*green*) and CP190 (*red*). Cells were grouped into cells with and without a detectable FLAG signal and analyzed for FLAG signal intensity and the number of CP190 intensity maxima using Fiji, a program for image analysis by ImageJ (Additional file 4: Table S2, Additional file 5: Table S3). **a** Examples of confocal micrographs. **b** Correlated FLAG-dSUMO signal after the indicated knockdown shows specific reduction of FLAG-dSUMO. Differences in corrected FLAG-dSUMO signal between mock knockdown and specific knockdown were analyzed by two-tailed Mann–Whitney *U* test. Significance level is defined as two-tailed asymptotic significance *p* < 0.05. **c** CP190-speckle number is significantly decreased in cells with high FLAG-dSUMO amount. Differences in number of CP190 intensity maxima between FLAG-dSUMO-positive and negative cells were analyzed by two-tailed Mann–Whitney *U* test. Significance level is defined as two-tailed asymptotic significance *p* < 0.05. Statistics were performed in SPSS^®^ (IBM^®^ SPSS^®^ Statistics 22)

 Nuclear foci of insulator proteins have been identified in different quality and quantity, depending on stress conditions of the analyzed cells as well as on microscopic resolution. Recently, we have refined the analysis of insulator speckles [43] and found more than 100 such foci, when analyzed by structured illumination microscopy (SIM) or 20 or less foci, when detected by confocal laser scanning microscopy (CLSM). Furthermore, we determined that insulator speckles are associated with long-distance chromatin contacts. In respect of the functional connection between CP190 and dSUMO as determined above, we asked whether dSUMO might have an effect on the number of insulator speckles. Therefore, we utilized the constitutively FLAG-dSUMO expressing cell clone to deplete these cells from both, dSUMO and FLAG-dSUMO, by double-stranded RNA. For this, we used two strategies, by targeting Smt3 (dSUMO) or by double knockdown of both components of the E1 SUMO-activating enzyme, Aos1 and Uba2. Both strategies should diminish the incorporation of SUMO and of FLAG-dSUMO into nuclear proteins in comparison with a negative control knockdown directed against luciferase. Cells were examined by antibody staining and CLSM analysis (Fig. 5a). In both strategies, nuclear staining with a FLAG-specific antibody resulted in a significant loss of staining after the specific knockdown (Fig. 5b). Co-staining with a CP190-specific antibody showed the expected speckled pattern for CP190. Examination of the speckle pattern by a program for automatic image analysis (Fiji), the number of CP190 intensity maxima could be determined (Fig. 5a, c). From our previous analyses [43], we predicted to find about 20 or less intensity maxima given the resolution of the CLSM microscope. We found and grouped the nuclei into two classes, one with most efficient knockdown without any detectable FLAG-dSUMO signal and one with an obvious FLAG-staining. Both knockdown strategies exhibited higher numbers of CP190 intensity maxima after full dSUMO depletion as compared to nuclei positive for FLAG-dSUMO (Fig. 5c). These differences were significant as analyzed by the two-tailed Mann–Whitney *U* test. The significance level is defined as two-tailed asymptotic significance *p* < 0.05.

Thus, we conclude that FLAG-dSUMO expression reduces the number of CP190-containing insulator speckles. As the overall CP190 content is not changed (see Fig. 2a), we propose that dSUMO causes the aggregation of insulator speckles such that the number of speckles is reduced. This, in turn, may increase the local concentration of insulator proteins and thereby improve the enhancer blocking activity, as shown above.

## Discussion

Functional consequences for SUMOylation in the context of chromatin insulation or enhancer blocking have been tested in the context of the *gypsy* insulator in *Drosophila*. It has been shown that the SUMO modification pathway antagonizes the insulator activity [29]. In contrast, SUMOylation of the insulator cofactor Mod(mdg4) improved the function of the *gypsy* insulator [34]. Based on our finding that components of the SUMOylation pathway improve the enhancer blocking function of the Fab8 insulator in an RNAi screen [22], we tested the enhancer blocking function of four different DNA sequences harboring binding sites for the insulator proteins dCTCF and CP190. Activity of each of these insulator sequences was impaired after depletion of enzymes involved in SUMO conjugation, indicating that SUMO conjugation improves the ability of insulator sequences to mediate enhancer blocking.

In order to identify insulator proteins, which, when bound by dSUMO, might explain SUMO-mediated improvement of enhancer blocking, we expressed a FLAG-dSUMO fusion protein. This strategy has been shown to help in the detection of SUMO-conjugated proteins [41]. Within a large fraction of SUMOylated proteins, we identified CP190 to be bound to FLAG-dSUMO. This is in agreement with previous observations [29, 34]. Apparently, dSUMO-conjugated CP190 does contribute to the functional effect of SUMO on insulation, as genome-wide binding of CP190 is significantly increased upon FLAG-dSUMO expression. In contrast, dCTCF binding to chromatin is not changed in agreement with our result that we could not detect dSUMO-conjugated dCTCF (not shown).

The cytological finding of insulator proteins clustering in the interphase nuclei resulted in a controversy on the in vivo existence and functional meaning of these foci. Early on, these structures have been termed insulator bodies, suggesting that these are sites of insulator action [9, 44–46]. This interpretation was challenged by the findings that insulator bodies are aggregated proteins not involved in insulation [34, 47]. Furthermore, osmotic stress causes the coalescence of diffusely distributed speckles into insulator bodies [48]. Insulator bodies have been analyzed in the context of SUMOylation, again with contradicting results. On the one hand, it was observed that dSUMO disrupts insulator bodies [29]. On the other hand, recruitment of specific insulator proteins into insulator bodies was found to require SUMOylation [34]. Recently, we re-analyzed the phenomenon of insulator factors clustered in nuclear foci [43]. We characterized more refined nuclear foci, termed nuclear speckles. These are structures marked by CP190, which are associated with chromatin sites of long-distance interaction [43]. These results together with the findings of this work may unify some of the apparent contradictions. Here we find that dSUMO does decrease the number of insulator speckles, but they are not destroyed. As the overall nuclear content of CP190 is not changed, the reduced number of CP190 speckles may be caused by fusion of CP190 speckles. Enhanced chromatin binding of SUMOylated CP190, as shown here, may also help in speckle fusion. Similarly, SUMO conjugation accomplishes other insulator (co-) factors to be recruited to insulator speckles [34]. Thus, SUMO-mediated factor recruitment and fusion of speckles may improve insulation and enhancer blocking function.

## Conclusions

Here we show that at least one of the cofactors of CTCF in *Drosophila*, CP190, is SUMOylated and that SUMOylation is required for optimal enhancer blocking. Depletion of any of the components of the enzymatic SUMOylation cascade impairs enhancer blocking. Mechanistically, SUMOylation is required for efficient genome-wide binding of CP190 to dCTCF.

## Methods

### Plasmids

Plasmids for dSUMO constitutive and inducible expression were used. pAW-FLAG-His-dSUMO contains a constitutive β-actin promoter. From this plasmid, we took FLAG-His-dSUMO to replace FLAG-HA-CP190 in the pRM-FLAG-HA-CP190 vector. pRM-FLAG-His-dSUMO has a CuSO4-inducible promoter. BamH1 was used to cut out FLAG-HA-CP190 and also FLAG-His-dSUMO from the corresponding plasmids. FLAG-His-dSUMO was inserted into the pRM empty vector by sticky end ligation.

### Cell culture

S2 wild-type cells, clones and clone pools were raised and cultured in Schneider’s Medium (Invitrogen; supplemented with 10% fetal bovine serum (FBS), 1% penicillin/streptomycin and glutamine). *Drosophila* S2 cells were transfected with the DNA plasmids for FLAG-dSUMO expression using the CaPO_4_ method and selected with puromycin (Gibco). S2 cell clones for luciferase reporter assay were transfected as described in before [22]. A cell clone for inducible FLAG-HA-CP190 expression was used as positive control for IP.

Synthesis of dsRNA and RNAi treatment was done as described on www.flyrnai.org (see Additional file 3: Table S1 for primer sequences). RNAi was performed for the analyses of CP190 speckles formation and of the functional luciferase reporter assay.

The luciferase reporter assay was performed as described previously [22].

### Antibodies

Primary antibody: α-IgG (polyclonal, mouse; GE Healthcare,); α-dCTCF N-terminal, α-dCTCF C-terminal (polyclonal, rabbit [10]); α-CP190 (polyclonal, rat [18, 47]); α-histone H3 (polyclonal, rabbit; Abcam, Ab-1791); α-β-Tubulin (monoclonal, mouse; DSHB Iowa, E7); α-FLAG M antibody (monoclonal, mouse; Sigma, F1804). Western Blot: 1:1000–1:20,000 dilution in PBS/0.1% Tween/3% milk; IF: 1:1000 dilution in 1% BSA; ChIP and FLAG-IP: 2–10 μl/IP. Secondary antibodies: α-mouse-IgG-HRP (Santa Cruz, sc-2055); α-rabbit-IgG-HRP (GE Healthcare, NA934V); α-rat-IgG-HRP (GE Healthcare, NA935V); α-mouse-IgG-Alexa 488 (Thermo Fisher, A32723); α-rat-IgG-Alexa 594 (Thermo Fisher, A-11007). Western Blot: 1:25,000 dilution in PBS/0.1% Tween/3% milk; IF: 1:25,000 diluted in 0.1% BSA.

### FLAG-IP

S2 cells were seeded 2 × 10^7^ cells/20 ml flask. Inducible cells were induced with 10 ng CuSO_4_ 24 h after seeding cells. After 48 h, the cells were harvested. Lysis was performed with SDS-Lysis-Buffer (50 mM Tris/HCl pH 7.5, 1% SDS, 10 mM NEM, 1 tablet PIC in ddH_2_O) (modified for *Drosophila* cells [49]) for 15 min at 4 °C. After that, the samples were diluted with IP buffer (20 mM Tris/HCl pH 7.5, 150 mM NaCl, 10% glycerol, 0.1% NP40, 1 mM DDT, 1 tablet cOmplete™ Protease Inhibitor Cocktail in ddH_2_O) [22]. Sonication with the Branson sonifier was necessary to reduce viscosity. Pre-clearing of the protein lysates was done by incubation with protein A/G agarose beads. IgG or FLAG-AB was incubated with the lysates for 2.5 h at 4 °C. The mix was then incubated with protein A/G agarose beads overnight by 4 °C. The beads were washed four times by IP buffer, incubated with 4 × Rotiload SDS loading dye and boiled at 100 °C for 15 min.

### ChIP

S2 cells were seeded 2 × 10^7^ cells/20 ml flask. Inducible cells were induced with 10 ng CuSO_4_ 24 h after seeding cells. After 48 h, they were harvested and counted. The ChIP was performed as described previously [21] with minor modifications in sonication. The cells were fixed in 1% formaldehyde (Calbiochem). Fixation was stopped by 0.125 M glycine (Merck). After washing cells with 1× PBS, lysis buffer (1% SDS, 10 mM EDTA, 50 mM Tris/HCl pH 8, 1 tablet cOmplete™ Protease Inhibitor Cocktail in ddH_2_O) was added to the cells and the DNA was fragmented with the Bio Ruptor sonicator (BioRad) for 2 × 10 cycles (30 s sonication, 30 s pause) within the ice-cooled sonicator. DNA was tested for fragment length of 200–500 bp on 1.5% agarose gel after phenol–chloroform extraction of DNA from chromatin. Chromatin samples were diluted 1:10 by dilution buffer (0.01% SDS, 1.1% Triton X-100, 1.2% EDTA, 16.7 mM Tris/HCl pH 8, 167 mM NaCl in ddH_2_O), with addition of glycerol for stability of the chromatin. 10% input samples were separated from prepared chromatin and were stored at −20 °C. Pre-clearing of chromatin was performed with protein A/G agarose beads (Millipore) for 45 min at 4 °C. IP with specific antibodies against CP190, dCTCF, FLAG and Histone H3 was performed at 4 °C. Mouse IgG was used as IP control. After 4 h, beads were added and the solution was further incubated at 4 °C. The beads were than washed with increasing salt concentration: low salt buffer (0.1% SDS, 1% Triton X-100, 2 mM EDTA, 20 mM Tris/HCl pH 8, 150 mM NaCl in ddH_2_O), high salt buffer (0.1% SDS, 1% Triton X-100, 2 mM EDTA, 20 mM Tris/HCl pH 8, 500 mM NaCl in ddH_2_O) and LiCl buffer (0.25% LiCl, 1% NP40, 1 mM EDTA, 10 mM Tris/HCl pH 8, 1% DOC in ddH_2_O), as well as 2× TE buffer (10 mM Tris/HCl pH 8, 1 mM EDTA). Clean up of DNA was performed with 100 µl Chelex 100 agarose beads (BioRad). Proteinase K digestion was done with 10 µg/ml of the enzyme during Chelex DNA extraction. DNA from 10% input samples were also extracted by the Chelex method [50].

### ChIP-sequencing

For sequencing, replicates of IPs had to be pooled. The pooled samples were concentrated by speed vac. 10 ng DNA of the S2 FLAG-dSUMO expressing pool induced (+CuSO_4_) and non-induced (−CuSO_4_) was collected for the corresponding IP with CP190, dCTCF and FLAG antibody. Input DNA was collected as control. Sequencing libraries were prepared with the NEBNext ChIP-seq Library Prep Reagent (New England Biolabs) according to manufacturer’s instructions. Cluster generation was performed using the cBot (Illumina Inc.). Sequencing was done on the HiSeq 2500 (Illumina Inc.) using TruSeq SBS Kit v3-HS (Illumina) for 50 cycles. Image analysis and base calling were performed using the Illumina pipeline v 1.8 (Illumina Inc.). Individual ChiP-seq data were validated extensively by specific qPCR. Raw and processed data have been deposited in the NCBI gene expression omnibus (GEO) under accession number (GSE96581). For validation of the ChIP-seq experiments, ChIP-qPCR was performed. 1 µl of ChIP-DNA was used for qPCR. Dilutions of the input (10, 1 and 0.1%) were used to determine a standard curve. The PCR was performed using specific 0.2 µM primers (Invitrogen) for CP190/dCTCF, CP190-only and negative sites. Primers (Suppl. Table S1) were partially chosen from the ChIP-seq experiment, but were also taken from previous publications [18, 21, 22]. qPCR was optimized for the BioRad CFX96 (96 well plate cycler) with an annealing temperature of about 60 °C.

### ChIP-sequencing and peak analysis

ChIP-seq reads were converted to fastq format and aligned to a precompiled dm3 reference index with BOWTIE [51]. Sequencing data were controlled for general quality features using FastQC. Unambiguously mapped and unique reads were kept for subsequent generation of binding profiles and calling of peaks using MACS and PeakRanger [52] using reads derived from sequencing of input DNA as control. Peaks were called at *p* < 10^−5^ and FDR <5%. All downstream analyses were done in R/BioConductor (http://www.bioconductor.org). Two peaks were determined to be overlapping in case they had a minimal overlapping interval of 1 bp.

In order to identify differentially bound regions, we collapsed all binding regions determined for a given factor under different conditions and extracted the read numbers mapping to these collapsed intervals. DESeq [53] was used to normalize between samples and in order to determine changes in occupancy and regions were ranked accordingly.

In case we were studying the overlap of multiple binding factors at once, we used the *multiinter* function of the BedTools suite [54] with the *cluster* option turned on. All intervals like binding peaks were annotated with respect to dm3 RefSeq annotations downloaded from UCSC genome browser repository.

Statistics for all meta-analyses were calculated using the Wilcoxon signed-rank test in R.

### Visualization of binding profiles

After extension of reads, continuous coverage vectors were calculated and normalized per million reads to account for differential library sizes. These data were used to collect data in windows of different sizes spanning features of interest (e.g., transcription factor peaks). The binding data were binned across binding sites in 50 bp windows, and the mean was calculated at each position in order to generate cumulative average binding profiles. For representation in genome browsers, profiles were additionally smoothed using kernel regression estimates. Data were visualized using the Gviz BioConductor package.

### Comparison to published data sets

For comparison with other transcription factor binding data we downloaded processed binding data from ChIP-experiments for dSUMO, Pita, ZIPIC, Polycomb, IbF1, CP190 und dCTCF [18, 19, 41, 42] were downloaded as SRA-archives from NCBI’s short read archive (SRA) and converted to fastq format using the *fastq*-dump tool of the SRA toolkit version 2.3.5. Reads were processed, and peaks were called as described above. Data sets used in this study were:

- GSM1015404, CP190; GSM1015406, Su(Hw); GSM1015408, dCTCF; GSM1015410, dCTCF; GSM1015410, Input [42].
- GSM1133262, Input; GSM1133264, CG8436 (IBF1) [19].
- GSM1278639, Beaf-32; GSM1278640, IgG [55].
- GSM1333827, Pc; GSM1333829, Input [41].
- GSM2042225, Pita; GSM2042228, Input [18].

### GO analysis of RNAi data

GO analysis was used to identify factors which correlate with one or more biological function. Here factors identified in the RNAi screen with a z score >2 were used for GEO analysis. Gene names were supplied into the GO term analysis tool GeneCoDis3 [56]. All factors, which were significant (*p* value <0.05) for one molecular function, were converted as a diagram in Microsoft Excel.

### Immune fluorescence staining

Immunostaining was performed as described [43]. S2 cells were seeded on coverslips and were RNAi treated for 4 days. Rat anti-CP190 [34] and mouse anti-Flag M2 were used.

Analyses of CP190 intensity maxima as well as determination of FLAG-dSUMO signal intensity were performed with Fiji, the image processing package of ImageJ. FLAG-dSUMO signal intensity measured by integrated density was background-corrected. Statistical analyses were carried out using SPSS^®^ (IBM^®^ SPSS^®^ Statistics 22). Differences in corrected FLAG-dSUMO signal between mock knockdown and specific knockdown were analyzed by two-tailed Mann–Whitney *U* test. Significance level is defined as two-tailed asymptotic significance *p* < 0.05. Differences in number of CP190 intensity maxima between FLAG-dSUMO-positive and negative cells were also analyzed by two-tailed Mann–Whitney *U* test. Significance level is defined as two-tailed asymptotic significance *p* < 0.05.

## Additional files

**Additional file 1: Figure S1.** dCTCF and CP190 peaks co-localize with previously identified dCTCF and CP190 binding sites. Peaks for dCTCF and CP190 were identified using MACS2 based on publicly available (GSE41354) ChIP-seq profiles published in Ong et al. [42] (PMID 24055367). Average binding of dCTCF as well as CP190 before and after expression of FLAG-dSUMO is shown across the known dCTCF (left) and CP190 (right) binding sites.

**Additional file 2: Figure S2.** Binding of CP190 is decreased after SUMO depletion. Binding of CP190 (red) after RNAi against GFP, against dSUMO (Smt3) and against both components of the E1 SUMO-activating enzyme, Aos1 and Uba2 (dark and pale red shading, respectively). ChIP-qPCR at CP190 example sites, in % of input. IgG was used as negative control (dark blue and pale shading). Sites without any binding CP190 (cg12054 neg. and lwr neg.) were used as control sites.

**Additional file 3: Table S1.** All primer sequences are listed.

**Additional file 4: Table S2.** Flag-SUMO and CP190 staining after luc or after Smt3 knockdown. Analyses of CP190 intensity maxima as well as determination of FLAG-dSUMO signal intensity were performed with Fiji, the image processing package of ImageJ. FLAG-dSUMO signal intensity measured by integrated density was background-corrected.

**Additional file 5: Table S3.** Flag-SUMO and CP190 staining after luc or after Aos/Uba2 knock down. Analyses of CP190 intensity maxima as well as determination of FLAG-dSUMO signal intensity were performed with Fiji, the image processing package of ImageJ. FLAG-dSUMO signal intensity measured by integrated density was background-corrected.

## Abbreviations

SUMO: small ubiquitin-like modifier
CP190: centrosomal protein 190 kDa
dCTCF: Dmel/CCCTC-binding factor
IBP: insulator-binding protein
Su(Hw): suppressor of hairy wing
BEAF-32: boundary element-associated factor of 32 kDa
Zw5: zeste white 5
GAF: GAGA-binding factor
ZIPIC: zink finger protein interacting with CP190
