# Supplementary figures and images for: Drosophila CP190- and dCTCF-mediated enhancer blocking is augmented by SUMOylation
Source: Epigenetics Chromatin. 2017 Jul 4;10:32. doi: 10.1186/s13072-017-0140-6 (PMC5496309; doi:10.1186/s13072-017-0140-6)

normalized binding

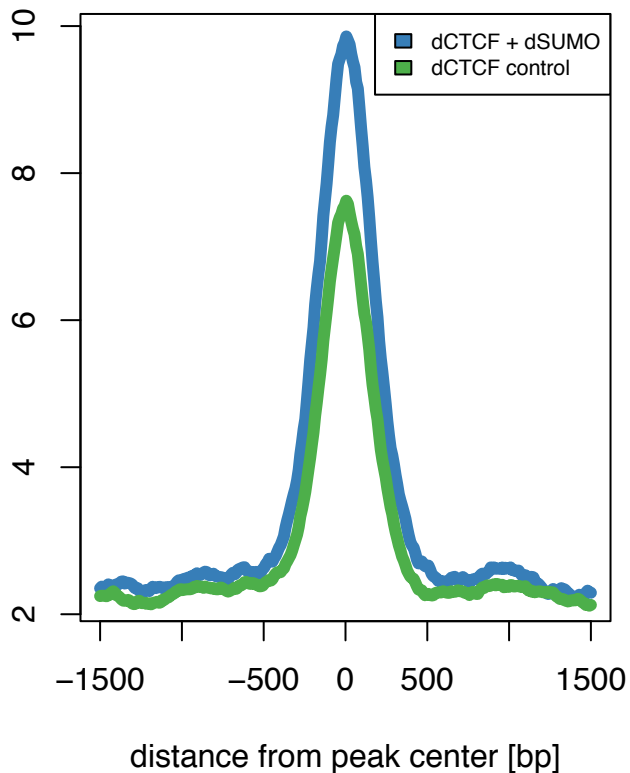

normalized binding

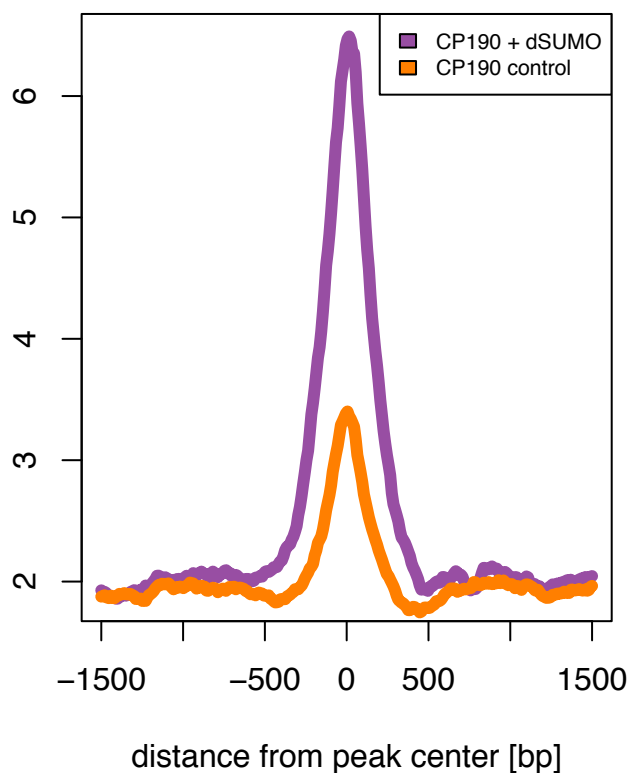

Supplement: Supplementary file 1 — Additional file 1: Figure S1. dCTCF and CP190 peaks co-localize with previously identified dCTCF and CP190 binding sites. Peaks for dCTCF and CP190 were identified using MACS2 based on publicly available (GSE41354) ChIP-seq profiles published in Ong et al. [42] (PMID 24055367). Average binding of dCTCF as well as CP190 before and after expression of FLAG-dSUMO is shown across the known dCTCF (left) and CP190 (right) binding sites. [file 13072_2017_140_MOESM1_ESM.pdf]

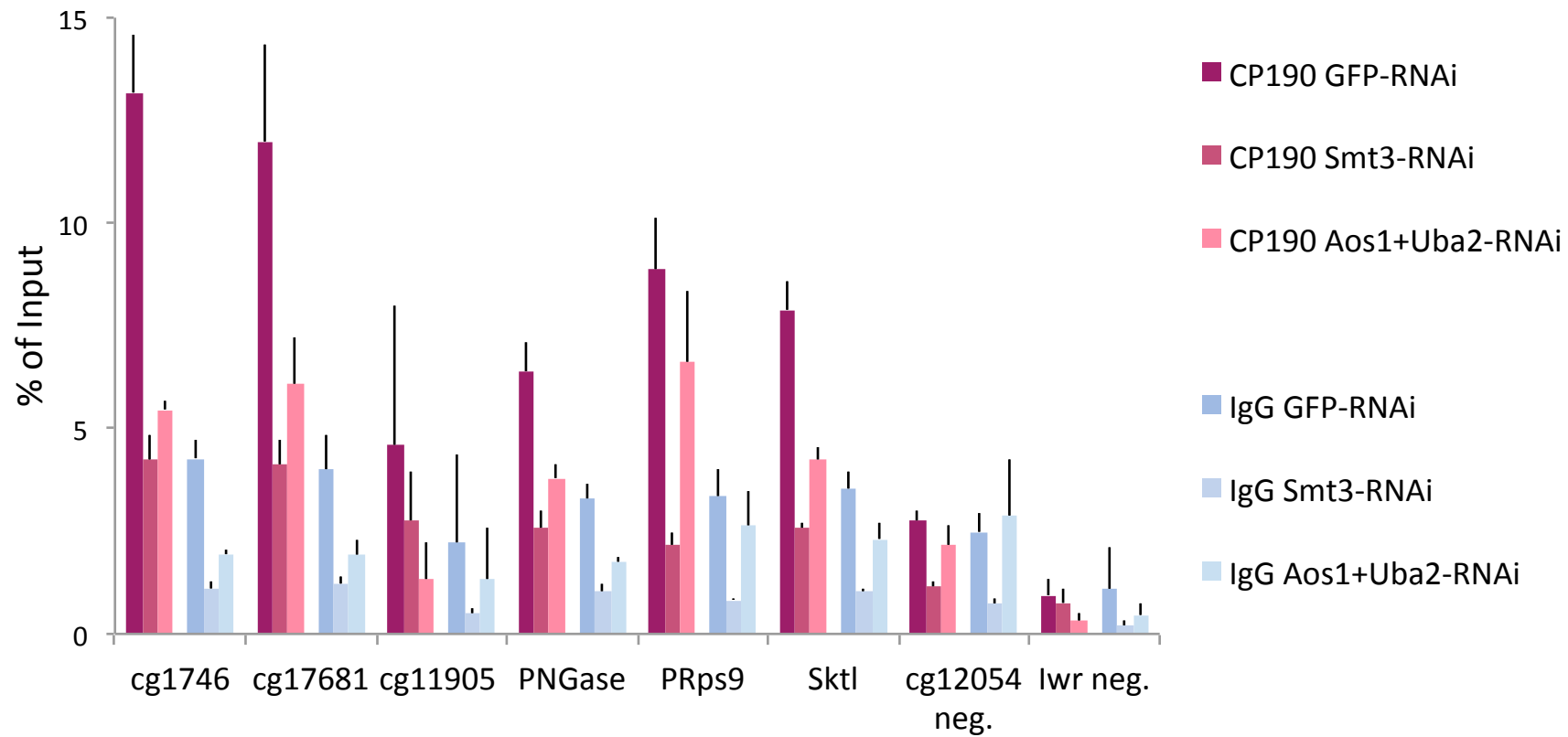

Supplement: Supplementary file 2 — Additional file 2: Figure S2. Binding of CP190 is decreased after SUMO depletion. Binding of CP190 (red) after RNAi against GFP, against dSUMO (Smt3) and against both components of the E1 SUMO-activating enzyme, Aos1 and Uba2 (dark and pale red shading, respectively). ChIP-qPCR at CP190 example sites, in % of input. IgG was used as negative control (dark blue and pale shading). Sites without any binding CP190 (cg12054 neg. and lwr neg.) were used as control sites. [file 13072_2017_140_MOESM2_ESM.pdf]
