# Supplementary material for: Drosophila CP190- and dCTCF-mediated enhancer blocking is augmented by SUMOylation
Source: Epigenetics Chromatin. 2017 Jul 4;10:32. doi: 10.1186/s13072-017-0140-6 (PMC5496309; doi:10.1186/s13072-017-0140-6)
Supplement: Supplementary file 3 — Additional file 3: Table S1. All primer sequences are listed. [file 13072_2017_140_MOESM3_ESM.docx]

**Supplemental Table S1: Primer sequences**

**RNAi-Primer**

| **Gen** | **DRSC Nr.** | **Gen Nr** | **[Bp]** | **Sequence** |
| --- | --- | --- | --- | --- |
| dCTCF |  |  | 338 | For 🡪GCCCGACATCAGTTCAAT  Rev 🡪GCACTTGAAGGATGGCTC |
| CP190 | MRC014_E10 |  | 510 | For 🡪TGCCGGGGACGATGATGACGATGAT  Rev 🡪CTCTTCTGGGCTGCGGCTGTATTTG |
| Aos1 | DRSC29634 | CG12276 | 316 | For 🡪TGGCAAAGTAGAACCCGAAG  Rev 🡪GTGAAGCTGCTGGATGACAA |
| Su(var)  2-10 | DRSC07721 | CG8068 | 505 | For 🡪CCATTTCCGTCTCTTCAGGA  Rev 🡪AGAAAGGCACCTCTTGGACG |
| lwr | DRSC27034 | CG3018 | 381 | For 🡪CAGTCCTTCTCCTCGTCCAG  Rev 🡪GACTGCTAGCGGAAATCCAG |
| Uba2 | DRSC11311 | CG7528 | 502 | For 🡪TGCATTTGGTCTAAGTCGGG  Rev 🡪GGGCAAGCTGTTCAACAAGT |
| velo | DRSC27497 | CG10107 | 420 | For 🡪CGGGGTTATGGTGGTGTTAC  Rev 🡪GAGGACTTCGTCTGCCTCAC |
| smt3 | DRSC03611 | CG4494 | 259 | For 🡪GCCACCAGTCTGCTGCTG  Rev 🡪TGACGAAAAGAAGGGAGGTG |
| T7 Seq |  |  | 2x23 | TAATACGACTCACTATAGGGAGA |

**ChIP-qPCR Primer (ChIP-Seq Validation)**

| **CP190 only sites** | **[Bp]** | **Sequence** |
| --- | --- | --- |
| cg1746 | 166 | For 🡪GAGCTGGGAAAAAGGACGAT  Rev 🡪CTATTGTGCAAGGGCAACCT |
| cg11905 | 249 | For 🡪GCTGTTCAGTGCCCCATACT  Rev 🡪CGGAGACCCACATCACTCTT |
| cg8776 | 163 | For 🡪TCCTCGAATGGGTTCACTTC  Rev 🡪TTCTGCTCGAAGCTTTCTGG |
| cg17681 5´GAF | 128 | For 🡪GTGGGACAGCTGACGAGAAT  Rev 🡪TGATCCATCCATCGCAAGTA |
| Sox102F (1) | 214 | For 🡪TAAACTCAGCCCTGCATTCC  Rev 🡪AAATCAAAAAGAGCTTCCTAAAAA |
| PNGase (Akt42) | 169 | For 🡪TGAAGACTGGAGAGCGGACT  Rev 🡪GGTACAGCATTTGGCGGTAT |
| RpS9 | 170 | For 🡪CCACGGAAACTAAACGGTCA  Rev 🡪TCCGCAATGGGATTGTATTT |
| sktl (insc) | 163 | For 🡪 GTGTTATCGTTTATCGCTTGC  Rev 🡪AACACTGCGCTTTGTTGCT |
| **dCTCF/CP190 sites** | **[Bp]** | **Sequence** |
| Ubx14 new | 183 | For 🡪CGAGTGCACCCTCCTAGTCT  Rev 🡪GATTTAGACTCCGCCCCTTT |
| CG31472 2 | 141 | For 🡪CATCGCCACCTATTGAGGAT  Rev 🡪CGTGCGCAAGTAGCAATG |
| Sbr CTS | 92 | For 🡪GCCATCTAGCGCCAGTACAT  Rev 🡪GTCGCCAACAGGAAACTGAC |
| EcR | 158 | For 🡪GCCTGAAACGGTTAAAGCAA  Rev 🡪CAACGGATGTGCGAGTAGAA |
| snRNA:U5:38ABb | 153 | For 🡪TCTGTCGGTTTTTGGTCTCC  Rev 🡪CACACGTCCTTTCCTGAAAAA |
| CR43437 | 155 | For 🡪GGTCCTAATTTCCGTGCTGA  Rev 🡪CACACGACGCATTTTGACAT |
| cg1124 | 199 | For 🡪GGCGTTGAATTGAGTCTGGT  Rev 🡪TTCAATCCGCGATACTGTGA |
| Hml 5´CTS | 210 | For 🡪 TGCACTGCTTGAACCGATAA  Rev 🡪TAGCCCCCTCTGCTGATATG |
| Fab8 new (UBX-65) | 317 | For 🡪 CGAACCATTGAAAGTTATCGA A  Rev 🡪CCTACGGCATTTTTCTGCTC |
| **Negative control** | **[Bp]** | **Sequence** |
| cg12054 | 160 | For 🡪CAAATGCAGGGACACCTTTT  Rev 🡪GCAAACCCTTTTGACCTTGA |
| Su(var)2-10 | 187 | For 🡪AGTCCGTGCAGGAACAAAAC  Rev 🡪ATGCTGTGTGTGTGGAGGAA |
| lwr | 188 | For 🡪GATCATGCGCAGCTTGTAGA  Rev 🡪TGTCCGGCATTGCTATTACA |
| Hml 5´-6´ctrl | 99 | For 🡪 GTGCAGTTTGCCCATATCAA  Rev 🡪ATTTTGCATCTTTCGCCATC |
